# Supplementary material for: Seasonal Single-Site Sampling Reveals Large Diversity of Marine Algal Toxins in Coastal Waters and Shellfish of New Caledonia (Southwestern Pacific)
Source: Toxins (Basel). 2023 Nov 3;15(11):642. doi: 10.3390/toxins15110642 (PMC10674433; doi:10.3390/toxins15110642)
Supplement: Supplementary file 1 [file toxins-15-00642-s001.zip › toxins-2669533-supplementary.pdf]

Supplementary Materials

Seasonal Single-Site Sampling Reveals Large Diversity of Marine Algal Toxins in Coastal Waters and Shellfish of New Caledonia (Southwestern Pacific)

Manoëlla Sibat, Tepoerau Mai, Simon Tanniou, Isabelle Biegala, Philipp Hess and Thierry Jauffrais

Table S1. Average amount (mean ± standard deviation, n = 3) with coefficient of variation (CV %) of toxin accumulated by SPATT bag during the two seasons after one week of exposure in Lemon Bay, Noumea.

Season	Toxicity in ng SPATT^{-1}				
	PTX2	AZA2	PnTx-G	OA	hYTX
Cold	3.0 ± 0.5	0.3 ± 0.1	0.6 ± 0.0	37.3 ± 4.2	12.6 ± 1.4
	19%	17%	4%	11%	11%
Warm	51.6 ± 10.1	0.6 ± 0.2	3.5 ± 0.7	37.2 ± 2.4	16.4 ± 2.6
	20%	28%	21%	7%	16%

Table S2. Quantitative results of the LC-MS/MS analysis obtained on the whole tissue of the shellfish collected in the bays of Noumea.

Sample Code	Toxicity in µg kg^{-1} of WT									
	hYTX	h45-OH-YTX	hCOOH-YTX	OA	PTX2	PTX2sa	AZA2	GYM-A	PnTX-G	BTX3
WT N°1	6	1.8	1.5	ND[1]	ND	ND	0.5	1	3.7	2.9
WT N°2	ND	ND	ND	ND	ND	ND	ND	2.3	8.3	ND
WT N°3	ND	ND	ND	ND	2.7	2.7	ND	1.3	1.0	ND
WT N°4	6.7	ND	0.6	0.6	0.0	2.2	1.3	1.5	7.0	ND
WT N°5	ND	ND	ND	ND	ND	ND	ND	8.0	16.0	ND
WT N°6	ND	ND	3.9	3.9	ND	ND	ND	8.4	0.8	ND
WT N°7	ND	ND	6.2	6.2	ND	2.6	0.2	0.7	22.6	ND
WT N°8	0.6	ND	ND	ND	ND	ND	ND	0.5	0.1	ND
WT N°9	ND	ND	ND	ND	ND	ND	ND	7.8	2.8	ND
WT N°10	ND	ND	ND	ND	ND	ND	ND	**22.6**	6.0	ND

Table S3. List of the selected MRM transitions and the MS parameters used for the detection of lipophilic toxins in negative ionization mode (**method 1**) and in positive ionization mode (**method 2 and method 3**).

**Ionization mode**	**Compound**	**Parent ion (*m*/*z*)**	**Fragment ion (*m*/*z*)**	**DP (V)**	**CE (eV)**	**CXP (eV)**
	OA & DTX2	803.4	113.1	-170	-92	-9
			255.1*		-62	-16
	DTX1	817.5	255.1*	-170	-68	-13
			113.1		-92	-5
	YTX	1141.4	1061.6*	-120	-48	-17
			855.6		-98	-19
	Homo-YTX	1155.5	1075.6*	-120	-48	-17
			869.4		-98	-19
**ESI-**	**45-OH YTX**	1157.5	1077.5*	-120	-48	-17
			855.5		-98	-19
	**45-OH homo YTX**	1171.5	1091.5*	-120	-48	-17
			869.4		-98	-19
	**COOH YTX**	1173.5	1093.5*	-120	-48	-17
			855.5		-98	-19
	**Homo COOH YTX**	1187.5	1107.5*	-120	-48	-17
			869.4		-98	-19
	Portimine	402.5	246.5*	100	58	10
			134.1		69	8
	GYM-A	508.4	490.2*	86	33	12
			162.4		69	10
	GYM-B/-C	524.4	506.4*	86	33	12
			162.4		69	10
	SPX-A / SPX desMe-C	692.7	444.3	121	53	26
	SPX-A		150.0*		69	8
	SPX desMe-C		164.2*		69	8
	SPX-B / SPX desMe-D	694.3	444.3	121	53	26
	SPX-B		150.0*		69	8
**ESI+**	***SPX desMe-D***		164.2*		69	8
	PnTX-G	694.4	440.2	125	60	10
			164.1*		80	8
	PnTX-A	712.4	440.2	125	60	10
			164.1*		80	8
	PtTX-A /-B /-C	831.5	458.5	125	60	10
			164.2*		80	8
	AZA 1	842.6	824.6*	116	41	12
			672.3		69	16
			654.4		69	16
	AZA2	856.6	838.6*	116	41	12
			672.4		69	16
			654.4		69	16
	AZA3	828.6	792.6*	116	41	12
			658.4		69	16
**ESI+**			640.4		69	16
	PTX2	876.6	823.3*	91	31	12
			805.6		37	12
	PTX2 sa	894.6	823.5*	91	31	12

			805.6		37	12
	PTX1	892.6	821.4*	91	31	12
			839.5		37	12
	PTX6	906.6	871.6*	91	31	12
			853.6		37	12
	BTX1	867.6	849.5*	151	21	22
			831.5		25	24
			813.6		27	20
	BTX2	895.6	877.5*	161	23	24
			859.6		29	24
			455.4		39	12
	BTX3	897.6	725.4*	151	29	18
			825.4		17	24
			807.5		19	24
	BTX-B5	911.6	893.6*	141	25	14
**ESI+**			875.6		27	14
			431.1		37	24
	BTX6	911.6	893.6*	120	30	20
			875.6		35	20
	BTX7	869.4	779.4*	120	25	20
			151.2		45	20
	BTX9	899.4	863.5*	120	35	20
			157.1		45	20
	Brevenal	657.4	639.3*	120	35	20
			579.3		35	20
			267.2		35	20

*transition used for quantification
